# Mutation N856K in spike reduces fusogenicity and infectivity of Omicron BA.1

**DOI:** 10.1038/s41392-022-01281-8

**Published:** 2023-02-22

**Authors:** Chunyun Sun, Huiyu Wang, Ji Yang, Desheng Kong, Yuning Chen, Haiyue Wang, Lingling Sun, Jianbo Lu, Min Teng, Liangzhi Xie

**Affiliations:** 1 Beijing Engineering Research Center of Protein and Antibody, Sinocelltech Ltd., Beijing, 100176 China; 2 Beijing Key Laboratory of Monoclonal Antibody Research and Development, Sino Biological Inc., Beijing, 100176 China; 3 Cell Culture Engineering Center, Chinese Academy of Medical Sciences & Peking Union Medical College, Beijing, 100005 China

**Keywords:** Cell biology, Microbiology, Molecular biology

**Dear Editor**,

COVID-19 lung pathology is characterized by interstitial pneumonia with cell-cell fusion-induced syncytia and extensive tissue damage.^1^ The correlation between viral fusogenicity and pathogenicity has been reported in SARS-CoV-2 variants.^2^ Compared with the previous Delta or D614G variants, Omicron BA.1 has been proven to be less fusogenic and pathogenic,^3^ potentially due to the high burden of mutations in spike, including 8 mutations in NTD, 15 in or adjacent to RBD, 2 in the furin-like cleavage motif (FL), and 6 in S2. However, the newly emerged Omicron sub-lineage BA.4/5 showed enhanced infectivity and fusogenicity compared to the BA.1/2.^4^ It seems that progressive mutational evolution of the spike in Omicron sub-lineages leads to increased transmissibility.^5^

To evaluate the fusogenicity of SARS-CoV-2 variants, we established a SARS-CoV-2 spike-mediated cell-cell fusion system using HEK293FT cells transiently expressing spike-GFP fusion protein (Supplementary Fig. 1a) as effector cells and Vero-E6 naturally expressing ACE2 as target cells. The spike-GFP fusion proteins of D614G, Delta or Omicron sub-variants were expressed on the cell membrane of effector cells (Supplementary Fig. 1b) with similar expression levels (Supplementary Fig. 2a). Large syncytia with strong fluorescence were observed when the D614G or Delta spike-GFP expressing cells were co-cultured with Vero-E6 after 48 h (Fig. 1a). Contrastingly, no obvious syncytia were formed in the Omicron BA.1 spike-GFP or GFP groups (negative control, NC) (Fig. 1a), even after 73 h (Supplementary Fig. 1c). Quantitative analysis showed that the cell fusion induced by Omicron BA.1 spike-GFP suffered a 9.2 (*p* < 0.0001) and 11.9-fold (*p* = 0.0003) reduction when compared to that induced by D614G and Delta spike-GFP, respectively (Fig. 1b), indicating a weakened capability of syncytia formation of the Omicron BA.1 spike. Interestingly, other Omicron sub-lineages, BA.2, BA.4/5, BA.2.12.1, and BA.2.75, maintained low fusogenic but caused more syncytia formation compared with BA.1 (Fig. 1b and Supplementary Fig. 1d).

**Fig. 1:**
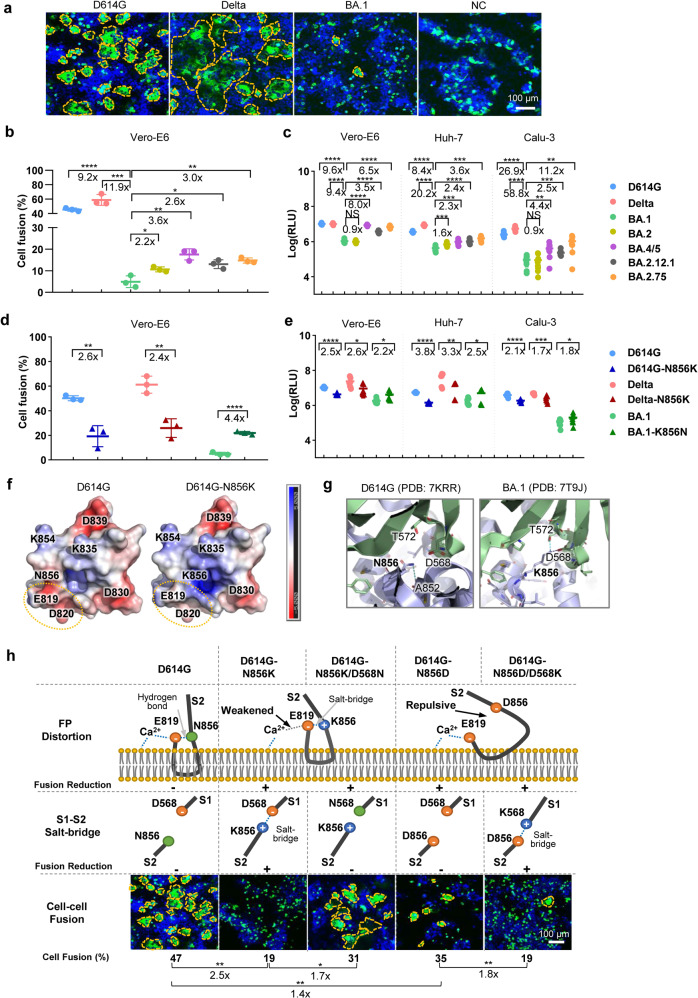
Mutation N856K reduces BA.1 virulence. **a** S-mediated cell fusion by different SARS-CoV-2 variants. Vero-E6 were used as target cells and co-cultured with HEK293FT effector cells expressing spike-GFP of different SARS-CoV-2 variants or single GFP (negative control, NC) for 48 h. Images were presented as merge of GFP and DAPI signals. Syncytia were highlighted by dotted yellow lines. Scale-bar: 100 μm. **b** Quantitative analysis of syncytia formation by SARS-CoV-2 variants. The experiments were performed in triplicate and the data were plotted as mean ± SD (*n* = 3). **c** PsVs infection signals of SARS-CoV-2 variants. Vero-E6, Huh-7, and Calu-3 cells were infected with the PsVs of SARS-CoV-2 variants at equal viral particles counts at 1E8 for 24 h. The experiments were performed in triplicate and data were plotted as mean ± SD (*n* = 9). Comparison was made between D614G, Delta, Omicron BA.2, BA.4/5, BA.2.12.1, BA.2.75, and Omicron BA.1, respectively. **d** Spike mediated cell–cell fusion of D614G and its N856K mutant, Delta and its N856K mutant, Omicron BA.1 and its K856N mutant. **e** PsVs Infection signals of D614G and its N856K mutant, Delta and its N856K mutant, Omicron BA.1 and its K856N mutant. **f** Electrostatic potential distribution on the solvent-accessible surfaces of the fusion peptides of D614G and N856K based on the PDB 7MY8 and a modeled structure, respectively. The electrostatic potential distribution was generated using the APBS Tools 2.1 in PyMOL. The surface potential representation has charge levels from −5kT/e (red) to +5kT/e (blue). The circles labeled with yellow lines indicated the binding region of Ca^2+^. **g** The interactions between residue 856 and its surrounding residues. The S2 subunits containing residue 856 in one protomer are in blue. The S1 subunits in another protomer are in green. Residues are shown as sticks. The hydrogen-bond and salt-bridge between residue 856 and other residues are shown as dashed lines in sky blue or dark blue, respectively. Superposition of the structures of the N856D/D568K mutant with that of the D614G S2 was superimposed and aligned. The predicted mutant structures were colored in yellow and the salt-bridge interaction between D856 and K568 was shown. **h** The schematic graphs of speculated structures in FP and S1/S2 interaction by structural analysis or modeling of D614 spike with residue 856 and/or 568 mutation designs and their speculated influences/experimental results on fusion activity. The panel of “FP Distortion” shows the proposed structure of fusion peptide (FP) when the type of residue 856 is asparagine (N), lysine (K), or aspartic acid (D). The panel of “S1-S2 Salt-bridge” shows that whether a salt-bridge was formed between residue 856 in S2 and residue 568 in S1 or not. The “Fusion Reduction” indicates the speculations for weakening fusion activity (+) or not (−). The panel of “Cell–cell fusion” indicated the experimental results of different D614G spike mutation constructs, which was showed by “cell fusion (%)”. Images were shown as merging of the GFP and DAPI signals, scale-bar: 100 μm. Quantitative analysis of cell fusion of different D614G spike mutants was shown below. The experiments were performed in triplicate and the data were plotted as mean (*n* = 3); Comparison was performed (1) among D614G and its N856 single amino acid mutants designed for verifying the FP disturbance speculation, and (2) between single amino acid mutants and double amino acid mutants designed for S1/S2 interaction speculation. Data analysis was performed by unpaired two-tailed Student’s *t*-test with or without Welch’s correction for all statistical analyses. *p < 0.05, **p < 0.01, ***p < 0.001, ****p < 0.0001, NS = *p* > 0.05

Spike-mediated infection activity was tested using a VSV based pseudovirus (PsV) assay. Similar spike expression levels were displayed on viral membrane of different SARS-CoV-2 variant PsVs (Supplementary Fig. 3). Those PsVs with normalized viral particles were used to infect Vero-E6, Huh-7, and Calu-3 (human airway epithelial cell line). The measured relative light unit (RLU) of the BA.1 PsV infected target cells was significantly lower than that of the D614G or Delta variant. Omicron BA.2 showed similar infectious activities to BA.1 PsV, while Omicron BA.4/5, BA.2.12.1, and BA.2.75, were more infectious than BA.1 (Fig. 1c). The tested virological feature of decreased cell fusion activity and lower viral replication of Omicron BA.1 is consistent with reported fusogenicity and infectivity of live BA.1 viruse.^3^

Omicron BA.1 contains a high burden of mutations scattered in different domains of the viral genome, especially in the spike protein. To identify domains in the spike protein responsible for the altered fusogenicity and infectivity of the Omicron BA.1, the Spike-based cell fusion assay and PsV infection assay were carried out with engineered spike proteins by domains swapping between D614G and BA.1 (Supplementary Fig. 4a). The results showed that the exchange of S2 regions between D614G and BA.1 severely reversed their fusogenicity and infectivity, while the exchange of NTD, RBD and FL regions had little effect (Supplementary Fig. 4b–d). Thus, the D614G spike-GFP mutants containing single site substitution in S2 region of Omicron BA.1 (N764K, D796Y, N856K, Q954H, N969K or L981F) were further evaluated. Interestingly, when compared with the parental D614G variant, a dramatic reduction in cell fusion was observed in the D614G spike containing the N856K substitution (Fig. 1d and Supplementary Fig. 5a), while minimum impact on fusogenicity was observed in the spike mutants with the other five substitutions (Supplementary Fig. 5b, c).

We speculated that the introduction of a N856K substitution into the Delta spike would also reduce its fusogenicity and other virological features, despite it being reported to have enhanced fusogenicity as a result of a P681R substitution.^6^ As expected, only small syncytia were observed in the fusion assay of the Delta-N856K spike compared to the naive Delta spike (Fig. 1d and Supplementary Fig. 5a). We then introduced the restorative mutation K856N into the Omicron BA.1 spike (BA.1-K856N) and found that the K856N substitution indeed increased the fusion of BA.1 (Fig. 1d and Supplementary Fig. 5a). Similarly, the infectivity of PsV containing D614G-N856K or Delta-N856K spike was significantly decreased compared to the corresponding parental D614G or Delta (Fig. 1e). However, PsV containing BA.1-K856N spike significantly increased the infection activity of BA.1 PsV (Fig. 1e). Similar to that of the BA.1-K856N, new sub-lineages of Omicron, BA.2, BA.4/5, BA.2.12.1 and BA.2.75 harbored K856N in S2 region showed slightly increased cell fusion and infection compared with that of Omicron BA.1 (Fig. 1b, c). Collectively, the above results suggested that N856K is a key mutation in the Omicron BA.1 variant responsible for the attenuated fusogenicity and infectivity.

However, the mechanisms by which N856K reduces the fusogenicity and infectivity of BA.1 need to be further explored. We found neither spike-GFP expression (Supplementary Fig. 2) nor ACE2 binding (Supplementary Fig. 6) was correlated with cell-cell fusion or PsV infection in Vero-E6, Huh7, or Calu-3. S1/S2 cleavage efficacy was also evaluated, and the N856K did not alter the S1/S2 cleavage ability of the native and the engineered spike proteins (Supplementary Fig. 7).

We then hypothesized two mechanisms by structural analysis. Firstly, the N856K may disturb the functional fusion peptide structure for membrane insertion. By electrostatic potential calculation, the negative charge potentials around E819 and D820 in the fusion peptide with N856K mutation were shown to be attenuated (Fig. 1f), which may lead to weaker electrostatic interactions and probably hinder the binding of E819 and D820 with Ca^2+^(Fig. 1h), an important regulator for cell fusion.^7^ To investigate the structural disturbance effect, we introduced N856D into the D614G. The D614G-N856D reduced cell fusion activity from 47 to 35% (1.4-fold, *p* = 0.0088) (Fig. 1h). Instead of a hydrogen bond between N856 and E819 in the WT fusion peptide (Supplementary Fig. 8a), the N856D substitution may introduce a repulsive interaction (Fig. 1h) that is disadvantageous for maintaining a wedge-shaped structure.^8^ Besides, a neutral amino acid substitution at N856, D614G-N856S, did not decrease the fusion activity (data not shown).

Secondly, the N856K may stabilize the pre-fusion spike conformation and encumber its post-fusion transformation required for the cell fusion. The N856 formed only one hydrogen bond with the backbone A852 in the same protomer in the D614G, while the K856 formed a hydrogen bond with T572^9^ and a salt-bridge with D568 in another S1 protomer in the Omicron BA.1^10^ (Fig. 1g), which could potentially prevent S1 shedding and hamper the conformational change of spike. To interrupt the salt-bridge between D568 and K856, an additional D568N mutation was introduced into the D614G-N856K. The double mutations (N856K/D568N) partially restored cell-cell fusion of D614G-N856K spike from 19 to 31% (1.7-fold, *p* = 0.0203), reaching a similar level as D614G-N856D (35%) (Fig. 1h). Then we introduced another double mutation D568K/N856D with switched residual types in Omicron into the D614G. The D614G-D568K/N856D modeling showed that a salt-bridge could be formed between K568 and D856 (Fig. 1h and Supplementary Fig. 8b). Consistent with the modeling analysis, D614G-D568K/N856D showed a further reduced fusion activity from 35 to 19% (1.8-fold reduction, *p* = 0.0040) compared to that of D614G-N856D, reaching a similar level as D614G-N856K (19%) (Fig. 1h).

In summary, N856K significantly reduced the fusogenicity and infectivity of Omicron BA.1. Reverse mutation K856N in Omicron BA.1 partially restored cell fusion activity (4.4-fold enhancement) and PsV infectivity (1.8 to 2.5-fold increasement), highlighting the importance of continuous monitoring and further investigations. Given the potential link between fusogenicity and disease severity of SARS-CoV-2 infection, it is useful and important to monitor the fusogenicity of future variants in addition to their transmissibility and vaccine immune evasion capabilities.

## Supplementary information

Supplementary Materials

## Data Availability

The data that support this study are available from the corresponding author upon reasonable request. Source data supporting the findings of this study are provided within the paper.

## References

[1] Bussani R (2020). Persistence of viral RNA, pneumocyte syncytia and thrombosis are hallmarks of advanced COVID-19 pathology. EBioMedicine.

[2] Zhang Y (2022). SARS-CoV-2 spike L452R mutation increases Omicron variant fusogenicity and infectivity as well as host glycolysis. Signal Transduct. Target Ther..

[3] Suzuki R (2022). Attenuated fusogenicity and pathogenicity of SARS-CoV-2 Omicron variant. Nature.

[4] Kimura I (2022). Virological characteristics of the SARS-CoV-2 Omicron BA.2 subvariants, including BA.4 and BA.5. Cell.

[5] Barozi, V., Edkins, A. L. & Bishop, Ö. T. Evolutionary progression of collective mutations in Omicron sub-lineages towards efficient RBD-hACE2: allosteric communications between and within viral and human proteins. *Comput. Struct. Biotechnol. J*. **20**, 4562–4578 (2022). 10.1016/j.csbj.2022.08.015 PMC9384468 35989699

[6] Saito A (2022). Enhanced fusogenicity and pathogenicity of SARS-CoV-2 Delta P681R mutation. Nature.

[7] Lai AL, Freed JH (2021). SARS-CoV-2 fusion peptide has a greater membrane perturbating effect than SARS-CoV with highly specific dependence on Ca2+. J. Mol. Biol..

[8] Koppisetti RK, Fulcher YG, Van Doren SR (2021). Fusion peptide of SARS-CoV-2 spike rearranges into a wedge inserted in bilayered micelles. J. Am. Chem. Soc..

[9] Cerutti G (2022). Cryo-EM structure of the SARS-CoV-2 Omicron spike. Cell Rep..

[10] Zhang J (2022). Structural and functional impact by SARS-CoV-2 Omicron spike mutations. Cell Rep..

