# Supplementary material for: Mutation N856K in spike reduces fusogenicity and infectivity of Omicron BA.1
Source: Signal Transduct Target Ther. 2023 Feb 22;8:75. doi: 10.1038/s41392-022-01281-8 (PMC9944258; doi:10.1038/s41392-022-01281-8)
Supplement: Supplementary file 1 — Supplementary Materials [file 41392_2022_1281_MOESM1_ESM.docx]

Supplementary Materials for

**Mutation N856K in Spike Reduces Fusogenicity and Infectivity of Omicron BA.1**

Chunyun Sun^1#^, Huiyu Wang^1#^, Ji Yang^1#^, Desheng Kong^1^, Yuning Chen^2^, Haiyue Wang^1^, Lingling Sun^1^, Jianbo Lu^1^, Min Teng^2^, Liangzhi Xie^1,2,3*^

1. Beijing Engineering Research Center of Protein and Antibody, Sinocelltech Ltd., Beijing 100176, China
2. Beijing Key Laboratory of Monoclonal Antibody Research and Development, Sino Biological Inc., Beijing 100176, China
3. Cell Culture Engineering Center, Chinese Academy of Medical Sciences & Peking Union Medical College, Beijing 100005, China

\# These authors contributed equally to this work

* Correspondence to: [LX@sinocelltech.com](mailto:LX@sinocelltech.com)

**This PDF file includes:**

Materials and Methods

Supplementary Figures 1 to 9

Materials and Methods

**Cell lines**

Vero-E6 cells were purchased from Chinese Academy of Medical Sciences Cell Bank (Cat.: GN017). Huh-7 cells were obtained from Chinese Culture Tissue Collection Center (CCTCC, China). HEK293FT cells used for transiently expressing spike were obtained from Chinese Academy of Medical Sciences. The human embryonic kidney HEK293T cell line (Cat.: CRL-11268) used for pseudovirus packaging was purchased from ATCC. Calu-3 cells were obtained from Shanghai Institutes of Biological Sciences, Chinese Academy of Sciences. HEK293FT, HEK293T, Vero-E6 and Huh-7 cells were grown in Dulbecco’s modified Eagle’s medium (DMEM) containing 10% (v/v) FBS. Calu-3 cells were grown in Minimum Essential Media (MEM) containing 10% (v/v) FBS.

**Reagents**

Paraformaldehyde (PFA, P6148) was purchased from Sigma-Aldrich. 4, 6-diamidino- 2-phenylindole (DAPI, 4083S) was purchased from Cell Signaling Technology. Luciferase Assay System (Cat.: E1501) and Passive Lysis 5× Buffer (Cat.: E1941) were purchased from Promega.

**Cell–cell fusion assay**

The establishment and detection of cell–cell fusion assay was performed as previously described^1^^,^^2^. In brief, Vero-E6 or Huh-7 cells were used as target cells and HEK293FT cells transfected with SARS-CoV-2 S-GFP (5 μg) of different variants and their domain swaps or amino acids mutations expression vectors verified by DNA sequencing using Sinofection (Sino Biological, Cat.: STF02) for 6 h were served as effector cells. Spike sequences were derived from wild type reference strain (isolate Wuhan-Hu-1) with D614G substitution (referred herein as D614G, EPI_ISL_406862), Delta (EPI_ISL_1999775), BA.1 (EPI_ISL_ 6640917), BA.2 (EPI_ISL_7190366), BA.2.12.1 (EPI_ISL_12087359.1), BA.2.75 (EPI_ISL_13392500.1), BA.4/BA.5 (EPI_ISL_11542270.1). Effector cells and target cells were co-cultured in DMEM containing 10% FBS for 48 h. After incubation, cells were fixed with 4% PFA at room temperature for 20 min and stained for nuclei with DAPI. The fluorescence images were recorded using a Leica SPE II confocal microscope with 20× objective lens. S-mediated cell-cell fusion was observed by the formation of multi-nucleated syncytia. Sixteen fields were automatically collected in each well by ImageXpress (Molecular Devices) to count the number of fused and unfused cells. The cell-cell fusion was calculated as the ratio of fused cell number and sum of fused/unfused cell number.

In order to observe the localization of spikes on membrane, effector cells overexpressing different spikes were cultured separately for 48h, then fixed with 4% paraformaldehyde, blocked with 5% BSA for 30min, co-incubated with APC-labeled ACE2-Fc recombinant protein (10 μg/mL) for 2 h at room temperature, and then washed with PBS for 3 times to remove the unbound protein. The images were taken by a Leica SPEII confocal microscope with 60× oil objective lens.

**Pseudovirus production in HEK293T adherent cells**

Pseudoviruses are replication-deficient vesicular stomatitis virus with its VSV-G protein gene replaced by a luciferase reporter gene in the genome. Construction of these pseudoviruses were described previously^3^. Briefly, expression vector pCMV3-SARS-CoV-2-S encoding spike protein of different variants and their domain swaps or amino acids mutations were verified by DNA sequencing before transfected into HEK293T cells and cultured in CO_2_ incubator for 16-24 hours. Replication-deficient vesicular stomatitis virus (VSV△G-Luc-G) was added into the above cell supernatant by a certain multiplicity of infection (MOI). After infection of cells, supernatant was discarded and cells were washed to remove the uninfected virus. Fresh medium was added for further culture. 24-48 hours after infection, virus was collected and filtered to discard cell debris. Pseudoviruses were concentrated and purified by ultrafiltration. The spike sequences corresponding to D614G (EPI_ISL_406862), Delta (EPI_ISL_1999775), BA.1 (EPI_ISL_ 6640917), BA.2 (EPI_ISL_7190366), BA.2.12.1 (EPI_ISL_12087359.1), BA.2.75 (EPI_ISL_13392500.1), BA.4/BA.5 (EPI_ISL_11542270.1) were downloaded from GISAID (<https://gisaid.org/>) and the DNA templates were obtained by whole gene synthesis or mutation.

**Quantitative RT-PCR detection of SARS-CoV-2 pseudovirus**

The pseudovirus was treated with benzonase at RT for 1 h to remove the free RNA or plasmid. Pseudovirus RNA was extracted using the QIAamp Viral RNA Mini Kit (250) (Qiagen, Cat.: 52906). cDNA library was obtained by two-step reverse transcription PCR using RT enzyme (Sino Biological, SRT-200T). The Vesicular Stomatitis Indiana virus genome (GenBank: J02428.1) was amplified by real time qPCR. The forward primer is 5'-GAATAAACATCGGGAAAGCAG-3' and the reverse primer is 5'-CCAAGTAGATACAAAGGCAACCA-3' protein, targeting a 145-bp-long fragment from a conserved region in the N gene. Standard pseudovirus stock solution was diluted and detected together with the samples. The viral load of the pseudovirus was calculated by substituting CT value in the standard curve.

***In vitro* pseudovirus infection assay**

To test the activity of PsVs, Vero-E6, Calu-3 or Huh-7 cells were seeded in 96 well plates at 30,000 cells/well and the same copies (1E8) of pseudovirus expressing SARS-CoV-2 spike protein were added the next day. After incubation for 24 h at 37℃ in 5% CO_2_ incubator, the culture supernatant was removed. Then 1× Passive lysis buffer (Promega, Cat.:E1941) was added at 50 μL/well to lyse the cells. Luciferase Assay System (Promega, Cat.:E1501) was used according to the product instructions and the relative light unit (RLU) was measured with a luminometer (Berthold Technologies, CentroXS^3^ LB 960 Microplate Luminometer) to evaluate luciferase activity. The background RLU was measured in the wells that received the cells but not the pseudoviruses.

**ELISA detection of Spike content on membrane of PsV**

The protein content of spikes on different pseudoviruses were measured by a specific antibody, namely mh676, which had broad spectrum binding with spikes of different SARS-CoV-2 sub-strains. Firstly, mh676 was diluted to 4 μg/mL and coated in 96 well-plates overnight at 4℃. The next day, the unbound antibody was washed 3 times. TBS buffer solution containing 2% BSA was used to block unspecific binding for 1 h at room temperature. Pseudoviruses of different SARS-CoV-2 strains were diluted to a particle number of 1E8 and incubated with mh676 at room temperature for 2 h. HRP-conjugated mh397 (Sino Biological, Cat.: 40150-D004-H) were diluted to 2 μg/mL and added to the washed plates and then incubated at room temperature for 1 hour. The plates were washed 3 times with TBS. Peroxidase substrate was added to each well and incubated for 30 minutes at 37°C. The reaction was terminated with 2 mM H_2_SO_4_, and the plates were read at 450 by an automatic microplate reader (BIOTEK, ELX808).

**Flow cytometry detection for GFP or ACE2 binding**

HEK293FT was transiently transfected with S-GFP for 54 h. Cells were digested and washed twice. 5×10^5^ cells were incubated with APC labeled ACE2-Fc protein (Sino Biological, Cat.:10108-H02H) at the indicated concentrations for 60 min. Cells were washed to remove unbound proteins and resuspended in buffer before detecting by flow cytometer (FACSCelesta, BD). FlowJo (V10) and GraphPad Prism 8.0 software were used to analyze the expression of GFP and the binding of ACE2.

**Western Blot for S1/S2 cleavage**

HEK293FT cells (1E7) were transfected with either 30 μg of a TMPRSS2 expression plasmid or an empty plasmid for 24 h. The TMPRSS2 or empty vector transfected HEK293FT cells were adjusted to a cell culture density of 6E+5 and transfected with 2 μg vectors encoding different spike proteins for 48 h. Cells were collected, washed, and lyzed in a lysis buffer (Beyotime Biotechnology, Cat.: P0013B) with a protease inhibitor cocktail (Beyotime Biotechnology, Cat.: P1006) after culture. Total protein content of each lysate was quantified by BCA assay and the lysates were diluted with a sample buffer (100 mM Tris-HCl, pH 6.8, 4% SDS, 12% β-mercaptoethanol, 20% glycerol and 0.05% bromophenol blue) and boiled for 10 min prior to gel separation. For the western blotting assay, an anti-SARS-CoV-2 S2 monoclonal antibody (Sino Biological, 40590-D001, working concentration at 1 μg/mL) and a rabbit anti-β-actin polyclonal antibody (Sino Biological, Cat.:109444-T32) were diluted at 1:10000 and used for protein detection while a horseradish peroxidase (HRP)-conjugated Goat anti-rabbit IgG polyclonal antibody (1:10,000) and a HRP-conjugated Goat anti-human IgG(Fc) polyclonal antibody (1:1000) were used as secondary antibodies. Chemiluminescence signals were detected using FluorChemE according to the manufacturer’s instructions. Bands were visualized using AlphaViewSA and the band intensity was quantified using Image J software (ImageJ).

**Structural Analysis**

The structure model of the spike with N856D/D568K mutation was constructed by homology modeling based on the D614G structure (PDB ID: 7KRR) using Discovery Studio 4.0 (Accelrys Software). The interaction diagrams of the interesting spike residues of Omicron, D614G and N856D/D568K mutant with their surrounding residues were analyzed by Discovery Studio 4.0 and displayed by the PyMOL Molecular Graphics System, Version 2.4 (Schrödinger, LLC). The structure model of the N856K FP was constructed by Discovery Studio 4.0 based on the WT FP structure (PDB ID: 7MY8). As a functional FP is contained in the S2 and only exposes its N-terminus, the charge of the FP C-terminus was assigned as neutral. The electrostatic potential distributions of WT and N856K FP structures were then generated using the APBS Tools 2.1 in PyMOL.

[**Statistical**](https://www.google.com/search?q=statistical+analysis&sa=X&ved=2ahUKEwjGsPX2tdj0AhX94zgGHTFKCxkQ7xYoAHoECAEQNw) **Analysis**

Data were plotted as mean±SD. Comparisons among groups were conducted by unpaired two-tail Student’s *t* test with (equal variances) or without (unequal variances) Welch’s correction. *P*-value of *p<0.05, **p<0.01, ***p<0.001, ****p<0.0001 were considered statistically significant. All statistical analyses were carried out using GraphPad Prism 8.0. All the measurements were taken from distinct samples.

**Supplementary References**

1	Zhu, L. *et al.* Double lock of a potent human therapeutic monoclonal antibody against SARS-CoV-2. *Natl. Sci. Rev.* **8**, nwaa297 (2021).

2	Wang, X. *et al.* Deletion of ER-retention motif on SARS-CoV-2 spike protein reduces cell hybrid during cell–cell fusion. *Cell Biosci.* **11**, 1-12 (2021).

3	Whitt, M. A. Generation of VSV pseudotypes using recombinant ΔG-VSV for studies on virus entry, identification of entry inhibitors, and immune responses to vaccines. *J. Virol. Methods* **169**, 365-374 (2010).

Supplementary Figures

**Supplementary Figure 1 Spike mediated cell fusion of SARS-CoV-2 variants**

(a) Schematic representation of the spike protein of SARS-CoV-2 variants. Constructs were expressed as either spike-GFP fusion proteins on the surface of HEK293FT cells and used for cell fusion assay or as spike proteins on pseudovirions for PsV assay. Domain designation: SP, signal peptide; NTD, N-terminal domain; RBD, receptor binding domain; FL, furin-like cleavage motif; FP, fusion peptide; TM, transmembrane domain; IC, intracellular tail.

(b) Spike-GFP constructs of D614G, Delta, as well as Omicron BA.1, BA.2, BA.4/5, BA.2.12.1, BA.2.75 and single spike (negative control, NC) were expressed on the membrane of HEK293FT effector cells and bind to the receptor ACE2. Top: GFP signal, middle: APC labeled recombinant ACE2-Fc protein (10 μg/mL), bottom: merge of GFP and APC. Scale-bar: 10 μm.

(c) Time-course kinetics of syncytia formation in the co-cultured HEK293FT effector cells expressing D614G or Omicron BA.1 spike-GFP with Vero-E6 at indicated time points. Scale-bar: 100 μm.

(d) Spike-mediated cell fusion of BA.2, BA.4/5, BA.2.12.1 and BA.2.75. Scale-bar: 100 μm.

**Supplementary Figure 2 Quantitative Analysis of the expression of spike-GFPs on HEK293FT effector cells**

(a) Expression of the spike-GFP of the D614G, Delta, as well as Omicron BA.1, BA.2, BA.4/5, BA.2.12.1 and BA.2.75 variants.

(b) Expression of the spike-GFP of the D614G, Omicron BA.1 and S2 swapping constructs.

(c) Expression of D614G and D614G -N856K, Delta and Delta-N856K, as well as Omicron BA.1 and BA.1-K856N.

(d) Expression of different single site mutations of the D614G spike-GFP.

The relative GFP mean fluorescence intensity (MFI) of each samples were normalized against untransfected HEK293FT cells (negative control, NC). The experiments were repeated three times. Data are presented as mean±SD (n=3). Statistical analysis were performed by unpaired two-tailed Student’s *t*-test with or without Welch’s correction. *p<0.05, **=p<0.01, ***=p<0.001, ****=p<0.0001, NS=p>0.05.

**Supplementary Figure 3 The protein content of spikes on different pseudoviruses**

PsVs of different SARS-CoV-2 variants were diluted to equal viral particles counts at 1E8 and the total content of spike on each PsV was detected by ELISA. The experiments were performed in triplicate and data were plotted as mean±SD (n=3). Statistical analysis were performed by unpaired two-tailed Student’s *t*-test with or without Welch’s correction. *p<0.05, **=p<0.01, ***=p<0.001, ****=p<0.0001, NS=p>0.05.

**Supplementary Figure 4 Cell fusion and PsV infection of chimeric Spikes with domain swapping from D614G or Omicron BA.1 variant**

(a) Diagram of chimeric spikes of D614G and Omicron BA.1 created by domains swapping. All constructs were expressed as spike-GFP for fusion assay or as spike on VSV pseudovirus particles for PsV assay.

(b) Cell–cell fusion of domain swapped chimeric spikes. The images were shown as merge of GFP and DAPI signals, scale-bar: 100 μm.

(c) Quantitative analysis of syncytia of domain swapped chimeric spikes. The experiments were performed in triplicate and the data were plotted as mean±SD (n=3).

(d) PsVs infection signals of domain swapped chimeric spikes. Vero-E6, Huh-7 and Calu-3 cells were infected with PsVs of D614G, Omicron BA.1 and their S2 swaps at equal particle counts at 1E8. The experiment was repeated three times and data were plotted as mean±SD (n=9).

Comparison was preformed between D614G or Omicron BA.1 and their domain swaps, and unpaired two-tailed Student’s *t*-test with or without Welch’s correction was used for all statistical analyses. *p<0.05, **=p<0.01, ***=p<0.001, ****=p<0.0001, NS=p>0.05.

**Supplementary Figure 5 Mutation N856K reduces D614G spike mediated cell-cell fusion**

(a) Cell–cell fusion of D614G and D614G -N856K, Delta and Delta-N856K, as well as Omicron BA.1 and BA.1-K856N. Images were shown as merge of GFP and DAPI signals, scale-bar: 100 μm.

(b) Cell–cell fusion of different D614G spike mutants with single point mutations from the Omicron BA.1 S2. Images were shown as merge of GFP and DAPI signals, scale-bar: 100 μm.

(c) Quantitative analysis of syncytia of those D614G spike mutants. Mean±SD (n=3) of three replicates was shown.

Comparison was preformed between D614G and its single amino acid mutants, and statistical analysis measured by unpaired two-tailed Student’s *t*-test with or without Welch’s correction. *p<0.05, **=p<0.01, ***=p<0.001, ****=p<0.0001, NS=p>0.05.

**Supplementary Figure 6 ACE2 binding activities to spikes of SARS-CoV-2 variants**

(a) ACE2 binding activities of D614G, Delta and Omicron BA.1, BA.2, BA.4/5, BA.2.12.1, BA.2.75 variants spike-GFPs. The APC-labeled ACE2-Fc was used for binding at a high concentration of 250 nM.

(b) ACE2 binding activities of D614G, Omicron BA.1 and their S2 swaps spike-GFP. The APC-labeled ACE2-Fc was used for binding at a high concentration of 250 nM.

(c) ACE2 binding curves of D614G and its N856K mutant, Delta and its N856K mutant, or Omicron BA.1 and its K856N mutant.

(d) ACE2 binding curves of D614G spike-GFP mutants with different single point mutation.

The relative mean fluorescence intensity (MFI Fold) of APC were normalized with untransfected HEK293FT cells (negative control, NC). The experiments were repeated three times. Data are presented as mean±SD (n=3). Statistical analysis measured by unpaired two-tailed Student’s t-test with or without Welch’s correction. *p<0.05, **=p<0.01, ***=p<0.001, ****=p<0.0001, NS=p>0.05.

**Supplementary Figure 7 S1/S2 cleavage efficiency of spike**

(a. b) Western blotting of D614G, Omicron BA.1 and their S2 swapped spike(a), Delta and its N856K mutant spike, as well as Omicron BA.1 and its K856N mutant spike (b) expressed on HEK293FT or TMPRSS2 expressing HEK293FT cells.

(c. d) The ratio S2/(S+S2) values of D614G, Omicron BA.1 and their S2 swapped spike(c), Delta and its N856K mutant spike, as well as Omicron BA.1 and its K856N mutant spike (d) based on data of a and b. The values were calculated by the signals of S2 against the sum signals of full-length S plus S2.

The experiments were repeated three times. Data are presented as mean±SD (n=3). Statistical analysis measured by unpaired two-tailed Student’s *t*-test with or without Welch’s correction. *p<0.05, **=p<0.01, ***=p<0.001, ****=p<0.0001, NS=p>0.05.

**Supplementary Figure 8 Structural analysis around residue 856.**

(a) Proposed wedge-shaped fusion peptide structure of D614G. The hydrogen bond between N856 and E819 is shown as dashed blue line.

(b) The interactions between D856 and its surrounding residues of N856D/D568K mutant. The modeled structure of the N856D/D568K mutant (shown as blue and green for S2 and S1, respectively) with the structure of the D614G (PDB: 7KRR) (shown as yellow) were aligned by S2. Residues are shown as sticks. The salt-bridge between D856 and K568 in N856D/D568K mutant are shown as dashed lines in dark blue.

**Supplementary Figure 9**

**The original and uncropped film of Western blots related to Supplementary Figure 7**
